# Point-of-Care Based Electrochemical Immunoassay for Epstein-Barr Virus Detection

**DOI:** 10.1155/2022/5711384

**Published:** 2022-05-30

**Authors:** Miao Yu, Ming Liu, Yuan Li

**Affiliations:** ^1^Department of Otorhinolaryngology, The First Hospital of China Medical University, No. 155 Nnajing Street Heping District, Shenyang 110000, Liaoning Province, China; ^2^Logistics Support Department, Shengjing Hospital of China Medical University, No. 36 Sanhao Street Heping District, Shenyang 110000, Liaoning Province, China; ^3^Department of Ophthalmology, The First Hospital of China Medical University, No. 155 Nnajing Street Heping District, Shenyang 110000, Liaoning Province, China

## Abstract

This work describes a label-free electrochemical immunosensor for the sensing of Epstein-Barr virus (EBV) with high sensitivity. First, a monolayer of 1,6-hexanedithiol (HDT) was fabricated on the screen-printed electrode surface by the interaction between sulfur atoms and SPE. AuNPs can be modified on the electrode by the Au-S bond formed between the HDT-free group and Au atom in AuNPs. Protein A is then adsorbed onto AuNPs. Several parameters were optimized. The optimum concentration of protein A is 0.6 mg/mL. The optimum immobilization time for protein A is 90 min. The optimum concentration of antibody is 80 *μ*g/mL. The optimum immobilization time for antibody is 90 min. Directional immobilization of EBV antibody is achieved by high affinity binding of protein A to the Fc segment of antibody. When antigen specifically binds to antibody, the formation of immune complexes blocks electron transfer of [Fe(CN)6]^4-/3-^ and is reflected in the detection of cyclic voltammetry/electrochemical impedance spectroscopy. The detection range is 1 pg/mL–l00 ng/mL with a LOD of 0.1 pg/mL. In addition, the proposed sensor exhibited an excellent antiinterference property.

## 1. Introduction

Epstein-Barr virus (EBV) is a double-stranded DNA virus with a genome length of about 172 kb. A large number of studies have shown that the incidence of nasopharyngeal carcinoma in high-incidence areas is closely related to EBV infection and has a crucial role in the occurrence of nasopharyngeal carcinoma [1, 2]. These include evidence that EBV DNA or RNA is expressed in almost all nasopharyngeal carcinoma cells. EBV-associated antibodies (e.g. VCA-IgA and EA-IgA) are detected in peripheral blood of patients with nasopharyngeal carcinoma. Large prospective cohort studies in recent years have shown that almost all patients with nasopharyngeal carcinoma are accompanied by a sharp increase in the titer of EBV-associated antibodies such as VCA-IgA [3, 4]. Once these EBV-related antibodies are positive and continuously elevated in the serum of normal people, the risk of nasopharyngeal cancer is greatly increased in the following years, and the risk of disease increases about 20 times [5]

In the case that the primary prevention of nasopharyngeal cancer has not made a breakthrough, improving the rate of early diagnosis of nasopharyngeal cancer and promoting early diagnosis and treatment of nasopharyngeal cancer also plays a crucial role. Clinical diagnosis and treatment practice show that the five-year survival rate of early nasopharyngeal cancer after treatment can reach more than 90% [6]. The five-year survival rate for advanced nasopharyngeal cancer is less than 50%. The clinical diagnosis of nasopharyngeal carcinoma is made by tissue biopsy under the electronic nasopharyngoscope, and then, pathological diagnosis is made by pathologist. Due to the large trauma of sampling, small tissue, and reliance on microscopic-macroscopic observation, about 10% of patients failed to be diagnosed at the first sampling [7]. In addition, nasopharyngeal biopsy strictly depends on the clinical experience of clinicians and the guidance of medical instruments such as the electronic nasopharyngoscope, which makes it difficult to carry out extensive screening application of nasopharyngeal cancer in high-incidence sites [8].

Analysis of plasma EBV DNA using PCR has been regarded as an effective method for EBV detection. However, the results vary between laboratories due to the lack of a standardized assay. Recently, anti-EBV antibody serological testing has been developed to be one of the routine screenings of NPC. However, conventional antibody detection based on enzyme-linked immunoassay (ELISA) suffers from low sensitivity and specificity. Therefore, there is an urgent demand for highly sensitive, specific, and stable methods to detect anti-EBV antibodies. Electrochemical biosensors convert biochemical information into an electronic readout, such as analyte concentration. Electrochemical biosensors play a positive role in bioanalysis because they enable sensitive, specific, and low-cost sensing of molecules [9–11]. Functional nanomaterials have excellent electrical conductivity and catalytic property. Therefore, using functional nanomaterials to construct electrochemical sensing interfaces can improve the sensitivity, selectivity, and stability of novel electrochemical biosensors [12–14]. The combination of nanomaterials and biometrics can amplify signal transduction and biometrics, thus achieving highly sensitive biosensing. In addition, the electrochemical biosensor is a hot research topic for portable bedside detection (POC) because of the advantages of portable and low-cost. Electrochemical immunosensor has the advantages of fast detection speed, high sensitivity, and low detection limit [15–17]. In recent years, it is often used for sensing of tumor biomarkers in cancer diagnosis and has attracted more and more attention of researchers. One of the main components of an electrochemical immunosensor is a layer of biosensors, usually antibodies, assembled on an electrode [18]. It will interact with the target molecule and affect the stability, specificity, sensitivity, and repeatability of the sensor greatly. As a recognition molecule, antibody gives the site of antibody-antigen reaction. Therefore, immobilization of antibody is very important [18–21]. A targeted antibody layer can greatly increase the binding ability of antigen and improve the performance of the detection system. *Staphylococcus* A protein (SPA) is a cell wall protein isolated from *Staphylococcus aureus*. It binds to the Fc segment of most types of immunoglobulins without affecting the antigen-binding site [22]. On the one hand, carbon atoms have a strong affinity for protein A, which forms a dense monolayer on the electro surface. On the other hand, there are four sites in the protein A molecule that bind to the Fc region of the immunoglobulin. When Fc of the antibody binds to protein A coated on the electrode, the Fab segment of the antigenic decision cluster is left exposed on the outer layer of the electrode, waiting for the antigen to react with it. Therefore, protein A is often used for the fixation of the antibody [23, 24].

This work describes a label-free immunosensor for high sensitivity sensing of EBV. First, a monolayer of 1,6-hexanedithiol (HDT) was fabricated on the SPE surface. AuNPs can be modified on the surface of the sensor electrode through the Au-S bond formed between the HDT-free group and the Au atoms. Protein A is then adsorbed to AuNPs. Directional immobilization of EBV antibody is achieved by high affinity binding of protein A to the Fc segment of antibody. The sensor can detect EBV with high sensitivity.

## 2. Experimental

Epstein-Barr virus (EBV) EBC antigen monoclonal antibody was purchased from Shanghai Lingchao Biotechnology Co., Ltd. Recombinant *Staphylococcus aureus* protein A (95%) was purchased from Hangzhou Newlong Biotechnology Co., Ltd. Chlorogold acid (48–50% Au basis), bovine serum albumin (BSA, 96%), potassium ferricyanide (99.95% metals basis), potassium ferricyanide (99.95% metals basis), ethanol (95%), concentrated sulfuric acid (H_2_SO_4_), Tween-20 (99%), potassium chloride (KCl, 99.8%), and H_2_O_2_ were purchased from Sinopac Reagent Co., Ltd. 1,6-Hexanedithiol (98%) was purchased from Aladdin Reagent Co., Ltd. Screen printing electrode (SPE) was purchased from Nanjing Youyun Biological Reagent Co., Ltd.

The synthesis process of AuNP was as follows: 1 mL of 0.01 M HAuCl_4_ and 1 mL 0.01 M sodium citrate solution were mixed in 36 mL water. 1 mL NaBH_4_ (0.1 M) was added to the above mixed solution under intense agitation and reacted for 4 h. NaBH_4_ was hydrolyzed completely, and Au seeds were obtained. Add 1 mL of seed solution to the mixture containing 0.25 mL HAuCl_4_ (10 mM), 0.05 mL NaOH (100 mM), and 0.05 mL ascorbic acid (100 mM) and shake gently. Then, 2 mL of the above solution was added to 18 mL of CTAB (containing 50 *μ*M NaI). Then, 2.5 mL HAuCl_4_ (10 mM), 0.50 mL NaOH (100 mM), 0.50 mL ascorbic acid (100 mM), and 90 mL CTAB (0.05 M) were added to form AuNP.

The SPE was immersed in ethanol containing 0.5% 1,6-hexanedithiol for 8 h to form sulfhydryl groups at the surface. After the clean process, the electrodes were placed in colloidal gold solution and treated at 4°C for 12 h. They were washed with pure water and blow-dried with nitrogen. AuNPs were covalently bonded to the electrode by Au-S bond to obtain AuNPs-modified SPE (AuNPs/HDT/SPE). The electrodes were then immersed in 0.6 mg/mL SPA solution for 90 min (SPA/AuNPs/HDT/SPE). The SPA-modified electrode was immersed in a PBS solution containing 80 ng/mL BEV antibody, incubated at 37°C for 90 min, and cleaned 3 times with cleaning solution to remove unbound antibody molecules on the electrode surface (anti-BEV/SPA/AuNPs/HDT/SPE). Finally, the unbound protein sites on the gold electrode were sealed with blocking solution. The electrochemical immunosensor for CEA detection was obtained by cleaning with cleaning solution and blowing dry with nitrogen (BSA/anti-BEV/SPA/AuNPs/HDT/SPE). The sensors were placed in EBV standard solutions of different concentrations, incubated at 37°C for 1 h to wash the nonspecific adsorbed EBV (BEV/BSA/anti-BEV/SPA/AuNPs/HDT/SPE), and then, the electrochemical test was conducted. All electrochemical measurements were conducted on a CHI 760 *E* electrochemical workstation. Cyclic voltammetry (CV) and electrochemical resistance spectrum scanning (EIS) were used for measurement. Potassium ferricyanide/potassium ferricyanide ([Fe(CN)6]^4-/3-^) was used as probe for measurement. All data recorded from the electrochemical working station have been submitted to the Origin for further analysis.

## 3. Results and Discussion

**Figure 1:**
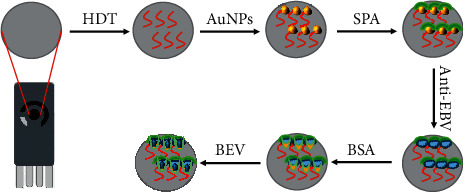
Scheme of electrochemical immunoassay fabrication.

This work describes a label-free immunosensor for the highly sensitive sensing of EBV. First, a single layer of HDT was fabricated on the SPE surface. Then, AuNPs were modified on the surface of the sensor electrode by the Au-S bond. Protein A then attaches to AuNPs. Targeted immobilization of EBV antibody was achieved by high affinity binding of protein A to the Fc segment of the antibody. The nonspecific sites on the electrode were sealed with bovine serum albumin before the immunosensor was used for EBV detection [25]. Schematic diagram of the immune sensor construction method is shown in Figure 1. When antigen is specifically bound to antibody, the formation of immune complexes blocks electron transfer and is reflected in the detection of CV/EIS.

**Figure 2:**
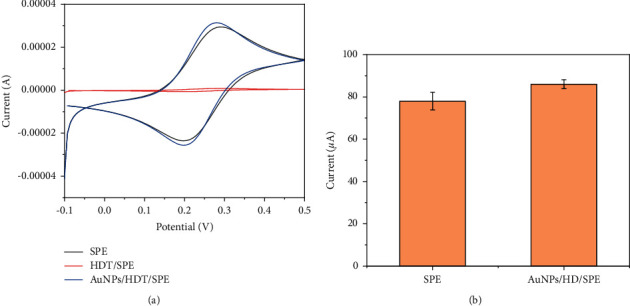
(a) Cyclic voltammetry of bare SPE, HDT/SPE, and AuNPs/HDT/SPE at [Fe(CN)6]^4-/3-^. (b) The mean and standard variance of redox current recorded by cyclic voltammetry scanning in [Fe(CN)6]^4-/3-^ at six electrodes before and after AuNPs modification (0.1 M PBS).

CV was used to characterize the electrochemical properties of AuNPs-modified electrodes.  Figure 2(a) shows the CV response of a bare SPE, HDT/SPE, and AuNPs/HDT/SPE in [Fe(CN)6]^4-/3-^. The bare SPE showed a reversible redox peak in [Fe(CN)6]^4-/3-^, and the oxidation current was different from the reduction current. After HDT modification, the redox peak almost disappeared because HDT formed a tight insulating monolayer on the electrode surface, which greatly hindered the electron transfer [26]. After immobilization of AuNPs on the SPE surface, the redox peak recovered completely. This further confirms that AuNPs successfully bonded to the SPE surface. The modification provides sufficient channel for electron transport as well as additional active sites for SPA adsorption [27].

Figure 2(b) shows the redox current and standard square difference recorded by CV of six electrodes in [Fe(CN)6]^4-/3-^ before and after AuNPs modification. It can be seen that AuNPs-modified electrodes not only have higher current but also show better uniformity [28]. This is also important for subsequent EBV detection.

**Figure 3:**
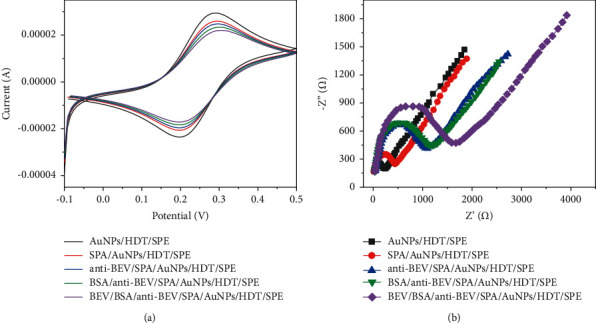
(a) CV curves and (b) EIS spectra of AuNPs/HDT/SPE, SPA/AuNPs/HDT/SPE, anti-BEV/SPA/AuNPs/HDT/SPE, BSA/anti-BEV/SPA/AuNPs/HDT/SPE, and BEV/BSA/anti-BEV/SPA/AuNPs/HDT/SPE in 5 mM [Fe(CN)6]^3-/4-^ (0.1 M PBS).

We used CV and EIS to detect the construction process.  Figure 3 shows the CV diagram and EIS diagram of the electrode at different stages. It can be seen from the EIS figure that the semicircle diameter decreases after the electrode surface is modified with AuNPs, indicating that AuNPs promotes electron transfer. When protein A was modified onto the AuNPs/SPE surface, the semicircle diameter increased. Meanwhile, the redox peak potential difference of redox electric pair [Fe(CN)6]^4-/3-^ increases, and the peak current decreases correspondingly [29, 30]. This indicates that protein A fixed on AuNPs blocks electron transfer and transfer on the electrode surface. After the antibody was bound to protein A, the semicircle diameter and redox peak increased significantly, while the peak current decreased [31]. The semicircle diameter also increased to a certain extent after BSA sealing, indicating that these biomolecules were successfully immobilized on the SPE surface. After incubating the antibody-modified electrode with 1 pg/mL BEV, the semicircle diameter was greatly increased, and the redox peak current was further reduced, which was due to the steric hindrance of the immune complex formed by BEV and its antibody [32]. The experimental data above show that the electrode modification method is effective.

**Figure 4:**
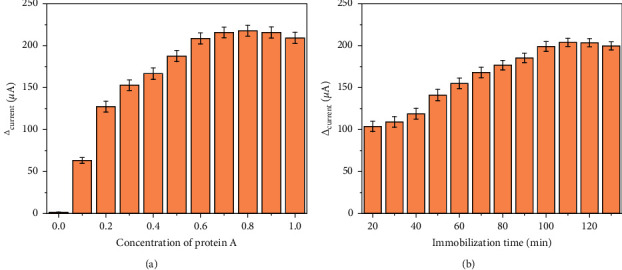
Effect of (a) the amount and (b) immobilization time of protein A in the sensor performance.

We investigated the effects of protein A concentration and immobilization time on current changes before and after immobilization. As shown in Figure 4(a), when the concentration of protein A varied from 0.1 mg/mL to 0.6 mg/mL, the amount of current change increased gradually. When the concentration of protein A was greater than 0.6 mg/mL, the change of current basically remained unchanged. Similarly, when protein A was immobilized for more than 90 min, the electrostatic adsorption of protein A on the electrode reached the saturation level [33], and the current change did not increase (Figure 4(b)). Therefore, 0.6 mg/mL was used as the concentration of protein A in the experiment, and 90 min was used as the fixed time of protein A.

**Figure 5:**
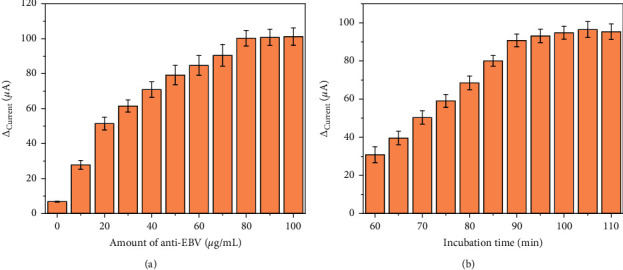
Effect of (a) the amount and (b) immobilization time of anti-EBV in the sensor performance.

The effect of antibody concentration on current change before and after immobilization was investigated in the range of 0–100 *μ*g/mL (Figure 5(a)). When the antibody concentration was less than 80 *μ*g/mL, the current change increased with the increase of antibody concentration. After excess of 80 *μ*g/mL, the change of current hardly increased, indicating that the antibody binding site of protein A was fully occupied. The experiment also explored the effect of antibody incubation time on antibody immobilization, as shown in Figure 5(b). When the incubation time is 90 min, the current change value reaches the maximum and does not change. Therefore, the optimal concentration and incubation time of immobilized antibody were 80 *μ*g/mL and 90 min, respectively.

**Figure 6:**
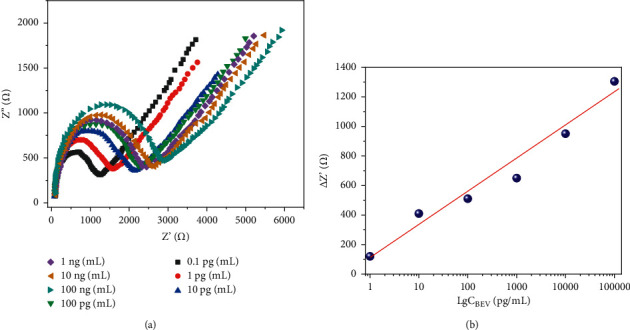
(a) EIS of fabricated sensor towards 1 pg/mL, 10 pg/mL, 100 pg/mL, 1 ng/mL, 10 ng/mL, and 100 ng/mL of EBV (0.1 M PBS). (b) Corresponding plots of concentration of EBV and ΔZ.

Under the optimal conditions determined above, we tested the response of the immunosensor to a series of EBV concentrations: 1 pg/mL, 10 pg/mL, 100 pg/mL, 1 ng/mL, 10 ng/mL, and 100 ng/mL.  Figure 6(a) shows Nyquist curves measured after the reaction of the immune electrode with the antigen. With the increase of antigen concentration, the amount of immune complex formed with the antibody on the electrode increases, and the obstruction to electron transfer is enhanced, and the diameter of the semicircle increases continuously [34].  Figure 6(b) shows the relationship between the change in semicircle diameter and the change in redox current and the logarithm of EBV concentration. The results show that in the range of 1 pg/mL–100 ng/mL, the change of semicircle diameter and redox current has a good linear relationship with the logarithm of EBV concentration. The detection limit of the sensor can be calculated as 0.1 pg/mL.  Table 1 provides the comparison of the proposed electrochemical sensor with previous reported sensors for EBV detection.

**Figure 7:**
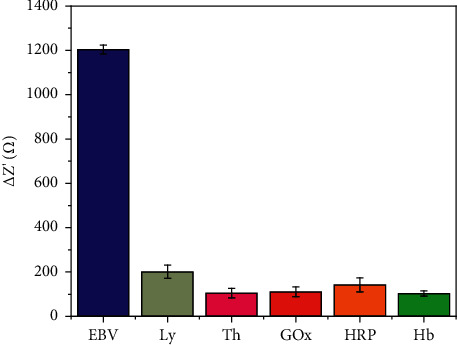
Antiinterference performance of the proposed electrochemical sensor.

The sensor's specific recognition performance was also tested. Lysozyme (Ly), thrombin (Th), glucose oxidase (GOx), horseradish peroxidase (HRP), and hemoglobin (Hb) were used as potential interference species. The results are shown in Figure 7. Due to the specificity of antigen-antibody, other interferers do not cause significant effects on the sensor.

## 4. Conclusion

This work describes a novel, sensitive, label-free electrochemical immunosensor for the sensing of EBV. First of all, AuNPs are bound to the electrode mediated by 1,6-hexanedithiol, which improves the electrical properties of the electrode and provides more active sites for protein adsorption. Protein A is then electrostatically adsorbed on the surface of the modified electrode to ensure directional immobilization of the antibody on the electrode. The immunosensor can detect EBV from 0.001 to 100 ng/mL. The detection limit was 0.1 pg/mL. Moreover, by changing the antibody immobilized on the sensor, it can be used to detect other biomarkers. In conclusion, the immunosensor provides an effective alternative to traditional immunoassay and has great potential in clinical application.

## Figures and Tables

**Table 1:** Comparison of different electrochemical sensors for EBV detection.

Sensor	Linear range	LOD	Reference
Polymer-based genosensor	3.78–756 *μ*M	17.32 nM	[35]
Microfluidic platform	300–10^7^ pg/mL	300 pg/mL	[36]
Metal-phenolic capsule	1 fM–1 nM	0.46 fM	[37]
DNA-based amplification	0.05–6.4 ng/mL	0.7 pg/mL	[38]
BEV/BSA/anti-BEV/SPA/AuNPs/HDT/SPE	1 pg/ml–100 ng/mL	0.1 pg/mL	This work

## Data Availability

The data used to support this study are available from the corresponding author upon request.
